# Low lipoprotein(a) concentration is associated with atrial fibrillation: a large retrospective cohort study

**DOI:** 10.1186/s12944-022-01728-5

**Published:** 2022-11-14

**Authors:** Junjie Tao, Xinlei Yang, Qingkai Qiu, Feng Gao, Wenchong Chen, Lijuan Hu, Yuan Xu, Yingping Yi, Hui Hu, Long Jiang

**Affiliations:** 1 Department of Cardiovascular Medicine, The Second Affiliated Hospital of Nanchang University, Nanchang, Jiangxi Province China; 2 Department of Clinical Medical, The Second Clinical Medical College of Nanchang University, Nanchang, Jiangxi Province China; 3 Department of Biobank Center, The Second Affiliated Hospital of Nanchang University, Nanchang, Jiangxi Province China; 4 Department of Nursing, Nanchang Medical College, Nanchang, Jiangxi Province China; 5 Department of Medical Big Data Center, The Second Affiliated Hospital of Nanchang University, Nanchang, Jiangxi Province China

**Keywords:** Atrial fibrillation, Lipoprotein(a), Cohort study, Relationship, Retrospective study

## Abstract

**Background and aims:**

The role of serum lipoprotein(a) [Lp(a)] levels in atrial fibrillation (AF) is still uncertain, especially in the Chinese population. Here, we aimed to elucidate the potential relationship between Lp(a) quantiles and AF.

**Methods:**

All data were collected through inpatients with electronic health records from the Second Affiliated Hospital of Nanchang University, Jiangxi Province, China. The propensity score matching (PSM) method was used to match control and case groups. Interactions between AF, Lp(a) quantiles, and other clinical indices were analyzed by logistic regression and stratified analysis. Statistical analyses were performed with IBM SPSS statistical software and R software.

**Results:**

From 2017 to 2021, 4,511 patients with AF and 9,022 patients without AF were 1:2 matched by the propensity score matching method. A total of 46.9% of the study group was women, and the baseline mean age was 65 years. The AF group exhibited lower median Lp(a) than the non-AF group (15.95 vs. 16.90 mg/dL; *P* < 0.001). Based on the Lp(a) quantiles, the study population was divided into four groups: Q1 (≤ 8.71 mg/dL), Q2 (8.71–16.54 mg/dL), Q3 (16.54–32.42 mg/dL) and Q4 (> 32.42 mg/dL). The AF prevalence of each group decreased from 34.2% (Q1) to 30.9% (Q4) (*P* < 0.001). Lp(a) quantiles 1–3 significantly increased AF to 1.162-fold (1.049–1.286), 1.198-fold (1.083–1.327), and 1.111-fold (1.003–1.231) in the unadjusted logistic regression model, respectively. In the adjusted model, Lp(a) < 32.42 mg/dL still showed a significant inverse association with AF. In the stratified analysis, Lp(a) levels in female patients exhibited a significant negative correlation with AF (OR of Q1: 1.394[1.194–1.626], *P* = 0.001). Age and hypertension did not affect the adverse correlation.

**Conclusion:**

Low circulating Lp(a) levels were associated with AF, especially in the female Han population, suggesting that Lp(a) may be useful for risk stratification of AF in female individuals.

**Supplementary Information:**

The online version contains supplementary material available at 10.1186/s12944-022-01728-5.

## Background

Atrial fibrillation (AF) has been increasingly considered a leading cause of cardiovascular events worldwide [1]. In the 2010 Global Burden Study, the prevalence of AF was 596 out of 100,000 men and 373 out of 100,000 women worldwide [2]. Current clinical evidence suggests that the overall AF ranges from 1 to 2% [3, 4]. Beyond that, approximately one-fourth to two-thirds of patients have transient or paroxysmal AF, which could cause the actual prevalence to be underestimated [5]. China's AF prevalence is increasing faster than the world average; approximately 3.9 million (2%) people over the age of 60 years were affected by AF in 2008 [6], and this number is expected to increase to 9 million by 2050 [7]. In general, patients aged 65 years or over are recommended to undergo opportunistic AF screening [8].

Lipoprotein(a) [Lp(a)] is a complex composed of apolipoprotein(a) [apo(a)] and apolipoproteinB-100 (apoB) linked through disulfide bonds. Elevated serum Lp(a) values have been confirmed as independent arteriosclerosis and coronary heart disease (CHD) risk factors [9]. Published guidelines and consensus statements have identified serum Lp(a) over 30 mg/dL as hyperlipoproteinemia(a) and recommended screening to lower the Lp(a)-mediated risk of cardiovascular events [10]. However, the mechanisms by which Lp(a) has a potential association with AF are not clear thus far. Most research did not find an obvious connection between AF and Lp(a). In a general population cohort study containing 109,440 individuals, elevated Lp(a) and most cardiovascular diseases had a strong positive correlation, while the relationship between Lp(a) and AF was not concordant [11]. In a multivariable Mendelian randomization study, high Lp(a) was weakly correlated with AF (OR and 95% CI, 1.001[1.000,1.002]). Of note, large-scale clinical studies that could verify the potential relationship are still lacking, especially with Asian participants.

Therefore, we conducted a retrospective study with a large sample size to investigate the potential AF-Lp(a) association in the Chinese population. Sex, CHD status, and other related factors that could influence the Lp(a)-AF relationship were also examined.

## Methods

### Study population

All data were collected through inpatients with electronic health records from the Second Affiliated Hospital of Nanchang University, Jiangxi Province, China. The ethics of this study were approved by the institutional review board of the Second Affiliated Hospital of Nanchang University.

### Definition and measurement of AF and other diseases

The patient's data were derived from their medical records; AF patients were identified as AF by a professional cardiologist based on the electrocardiogram. The diagnostic criteria for AF were no apparent P wave repetition, and irregular RR intervals were detected on electrocardiography (ECG) [12]. We defined the first diagnosis day of AF as the onset day. A CHD diagnosis was made when satisfying at least one coronary artery or its major branch had stenosis > 50% on coronary angiography [13].

### Clinical and laboratory analyses

General information was collected, including age, sex, body mass index (BMI), alcohol consumption, smoking, systolic blood pressure (SBP), and diastolic blood pressure (DBP). Measurement of Lp(a) concentration: After fasting for over 8 h, we collected the patient’s fresh serum and used the Lp(a) Assay Kit (Latex-Enhanced Immunoturbidimetric Method, Beijing Antu Inc, China, LOT: 10723C11) to measure Lp(a) levels, where 0 to 3000 mg/dL is the standard reference range for Lp(a). The experimental principle of the Lp(a) kit is as follows: Lp(a) reacts with the mouse anti-human lipoprotein(a) monoclonal antibody present on the latex particles. Then, the agglomeration of latex particles increases the turbidity in the solution. The calibration curve of absorbance and concentration was established by measuring a series of calibrators. By comparison with the established calibration curve, the Lp(a) concentration of the samples can be identified.

The laboratory data of albumin, apolipoprotein (Apo(A)), apolipoprotein B (Apo(B)), blood glucose, C-reactive protein (CRP), creatinine, high-density lipoprotein cholesterol (HDL-C), homocysteine (HCY), low-density lipoprotein cholesterol (LDL-C), total cholesterol (TC), triglyceride (TG), and uric acid were recorded.

### Statistical analyses

Statistical analyses were performed with IBM SPSS statistical software, version 21.0 (SPSS Inc., Chicago, Illinois), and R software, version 4.1.1. The level of significance was 0.05. PASS, version 15.0, was used to estimate the required sample size.

The AF group and non-AF control group were 1:2 matched using the PSM method for balancing covariates. PSM is a statistical method used to ensure that study participants are comparable on clinical measures and reduce bias. PSM determines whether the variable is a responder or a confounder when creating a regression model. The propensity scores for each subject were estimated to range from 0 to 1, indicating how the subjects should be divided into treatment groups.

In the baseline analysis, the median and quantile deviation were used to describe the continuous data because the Kolmogorov‒Smirnov test showed that all of the data were skewed (*P* < 0.05). For categorical variables, the number and percentage of cases were used to describe the data. All patients were equally sent into four groups by Lp(a) quantiles: quantile 1 (Q1), under 8.71 mg/dL; quantile 2 (Q2), 8.71–16.54 mg/dL; quantile 3 (Q3), 16.54–32.42 mg/dL; and quantile 4 (Q4), higher than 32.42 mg/dL. Binary logistic regression models were used to evaluate the correlation, and the risk prediction equation and odds ratio with the confidence interval for each factor were calculated by SPSS. Model 1 is the unadjusted analysis model. Model 2 uses BMI and SBP for adjustment. Model 3 was further adjusted for TG, CRP, HCY, blood glucose, and statin status. Subgroup analyses were designed to evaluate the influences of age (≤ 65 years and > 65 years), sex (men and women), CHD, hypertension status, and T2DM status on the relationship between Lp(a) and AF.

## Results

### Patient characteristics

**Fig. 1:**
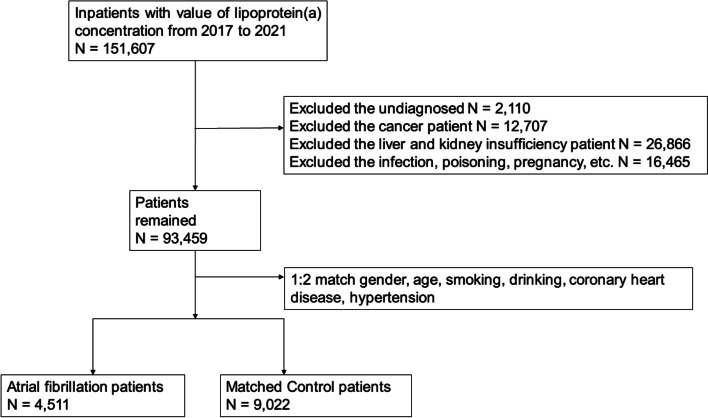
Flow chart of this Study

From January 2017 to July 2021, a total of 151,607 inpatients were included. Among them, 2,110 patients with unknown AF status, 12,707 cancer patients, 26,866 kidney dysfunction patients, and 16,465 patients with pregnancy, infection, and poisoning were excluded. The control group was 1:2 matched with the propensity score matching (PSM) method by the following items: sex, age, smoking, drinking, CHD, and hypertension status. Finally, the case group included 4,511 AF patients, and the control group included 9,022 non-AF patients (Fig. 1).

The baseline characteristics classified by the diagnosis of AF are summarized in Table 1. The baseline median age was 65 years. A total of 49.6% of patients in the AF group were women. The distribution of serum Lp(a) in the 13,533 participants was skewed (Supplementary Fig. 1). The AF group exhibited a lower median Lp(a) value (15.95 vs. 16.90, *P* < 0.001). Moreover, the control group exhibited higher Apo(A), Apo(B), TC, LDL-C, and creatinine levels (Table 1). All correlation indices between Lp(a) and the other clinical attributes are listed in Supplementary Table 1.

**Table 1:** Baseline profiles classified by case group or control group

Characteristics	Control group	AF group	*P* value (ASD)
**NO. (%)**	9022 (66.7)	4511 (33.3)	-
**Demographic data**			
Women, n(%)	4225 (46.83)	2123 (47.06)	0.798 (0.46%)
Ages (years)	69 [61,76]	69 [61,76]	0.993( 0.10%)
BMI	23.56 [21.45,25.55]	23.62 [21.55,25.78]	0.021
Smoker, n(%)	1300 (14.40)	642 (14.23)	0.781 (0.50%)
Alcohol taking status, n(%)	1161 (12.86)	583 (12.92)	0.928 (0.16%)
SBP (mmHg)	144 [130,159]	138 [125,152]	< 0.001
DBP (mmHg)	84 [76,92]	85 [76,94]	0.013
**Medical history**			
Ischemic stroke, n(%)	1647 (18.25)	1129 (25.03)	< 0.001
Type 2 diabetes mellitus, n(%)	1908 (21.14)	728 (16.14)	< 0.001
Coronary heart disease, n(%)	2431 (26.94)	1215 (26.93)	0.989 (0.24%)
Hypertension, n(%)	5018 (55.62)	2505 (55.53)	0.922 (0.17%)
**Medication on admission**			
β-receptor antagonists, n(%)	2357 (26.12)	3033 (67.24)	< 0.001
Statin, n(%)	4312 (47.79)	2239 (49.63)	0.043
**Laboratory values**			
Lp(a) (mg/dL)	16.90 [8.80,33.29]	15.95 [8.57,30.70]	< 0.001
LDL-C (mmol/L)	2.70 [2.12,3.29]	2.35 [1.84,2.93]	< 0.001
ApoA (mmol/L)	1.18 [0.98,1.41]	1.09 [0.92,1.32]	< 0.001
ApoB (mmol/L)	0.87 [0.70,1.07]	0.77 [0.61,0.96]	< 0.001
HDL-C (mmol/L)	1.18 [0.97,1.44]	1.11 [0.93,1.33]	< 0.001
TC (mmol/L)	4.63 [3.90,5.40]	4.09 [3.41,4.81]	< 0.001
TG (mmol/L)	1.27 [0.92,1.86]	1.09 [0.81,1.55]	< 0.001
HCY (μmol/L)	13.27 [11.13,16.18]	13.78 [11.31,17.20]	< 0.001
Albumin (g/L)	39.48 [36.58,42.48]	37.54 [35.03,40.22]	< 0.001
CRP (mg/L)	3.67 [2.15,8.78]	4.03 [2.21,9.96]	< 0.001
Blood glucose (mmol/L)	5.58 [4.90,6.81]	5.17 [4.62,6.14]	< 0.001
Uric acid (μmol/L)	338.03 [278.77,408.09]	377.58 [310.25,458.53]	< 0.001
Serum creatinine(μmol/L)	72.00 [60.63,85.64]	78.00 [65.21,91.99]	< 0.001

Continuous data are presented as the median [25, 75%]. The differences in the covariates included in PSM were evaluated by the *P* value and the absolute standardized mean difference (ASD)

*ApoA* Apolipoprotein A, *ApoB* Apolipoprotein B, *BMI* Body mass index, *CHD* Coronary heart disease, *CRP* C-reactive protein, *DBP* Diastolic blood pressure, *HDL-C* High-density lipoprotein cholesterol, *HCY* Homocysteine, *LDL-C* Low-density lipoprotein cholesterol, *Lp(a)* Lipoprotein (a), *SBP* Systolic blood pressure, *TC* Total cholesterol, *TG* Triglyceride

### Lp(a) and AF

Among the four groups stratified by Lp(a) quantiles, the incidence of AF was 34.2% (Q1), 34.9% (Q2), 33.2% (Q3) and 30.9% (Q4) (P for trend < 0.001). In the primary unadjusted model, Lp(a) quartiles 1–3 increased the AF 1.162-fold (95% CI: 1.049–1.286), 1.198-fold (1.083–1.327), and 1.111-fold (1.003–1.231), respectively (Table 2). In Model 2 adjusted for SBP and BMI, the OR with 95% CI of quantiles 1–3 exhibited 1.152 [1.040–1.277], 1.192 [1.076–1.321], and 1.119 [1.009–1.240], respectively (Table 2). In addition, all three Lp(a) quartile groups still showed a significantly increased AF in Model 3 after adjusting for TG, CRP, HCY, blood glucose, and statin status, plus Model 2. The ORs with 95% CIs of quantiles 1–3 were 1.241 [1.117–1.378], 1.226 [1.105–1.360], and 1.120 [1.008–1.243], respectively (Table 2).

**Table 2:** Odds ratios (95% confidence intervals) for the regression analysis of AF and Lp(a) quantiles

	**Model1**	**Model2**	**Model3**
Lipoprotein(a) quantiles			
Q1 [≤ 8.71 mg/dL]	1.162 (1.049–1.286) **	1.152 (1.040–1.277) **	1.241(1.117–1.378) **
Q2 [8.71—16.54 mg/dL]	1.198 (1.083–1.327) **	1.192 (1.076–1.321) **	1.276(1.105–1.360) **
Q3 [16.54—32.42 mg/dL]	1.111 (1.003–1.231) *	1.119 (1.009–1.240) *	1.120(1.008–1.243) *
Q4 [> 32.42 mg/dL]	Reference	Reference	Reference

Values are expressed as ORs (95% confidence intervals)

*AF* Atrial fibrillation, *Lp(a)* Lipoprotein(a), *BMI* Body mass index, *SBP* Systolic blood pressure, *TG* Triglyceride, *CRP* C-reactive protein, *HCY* Homocysteine

Significant interactions (*P* < 0.05) of Lp(a) quartiles and AF are marked with *; more significant interactions (*P* < 0.01) are marked with **

Model 1: unadjusted model for Lp(a) quartiles and AF

Model 2 adjusted for BMI, SBP, plus Model 1

Model 3 adjusted for blood glucose, CRP, HCY, statin status, TG, plus Model 2

**Fig. 2:**
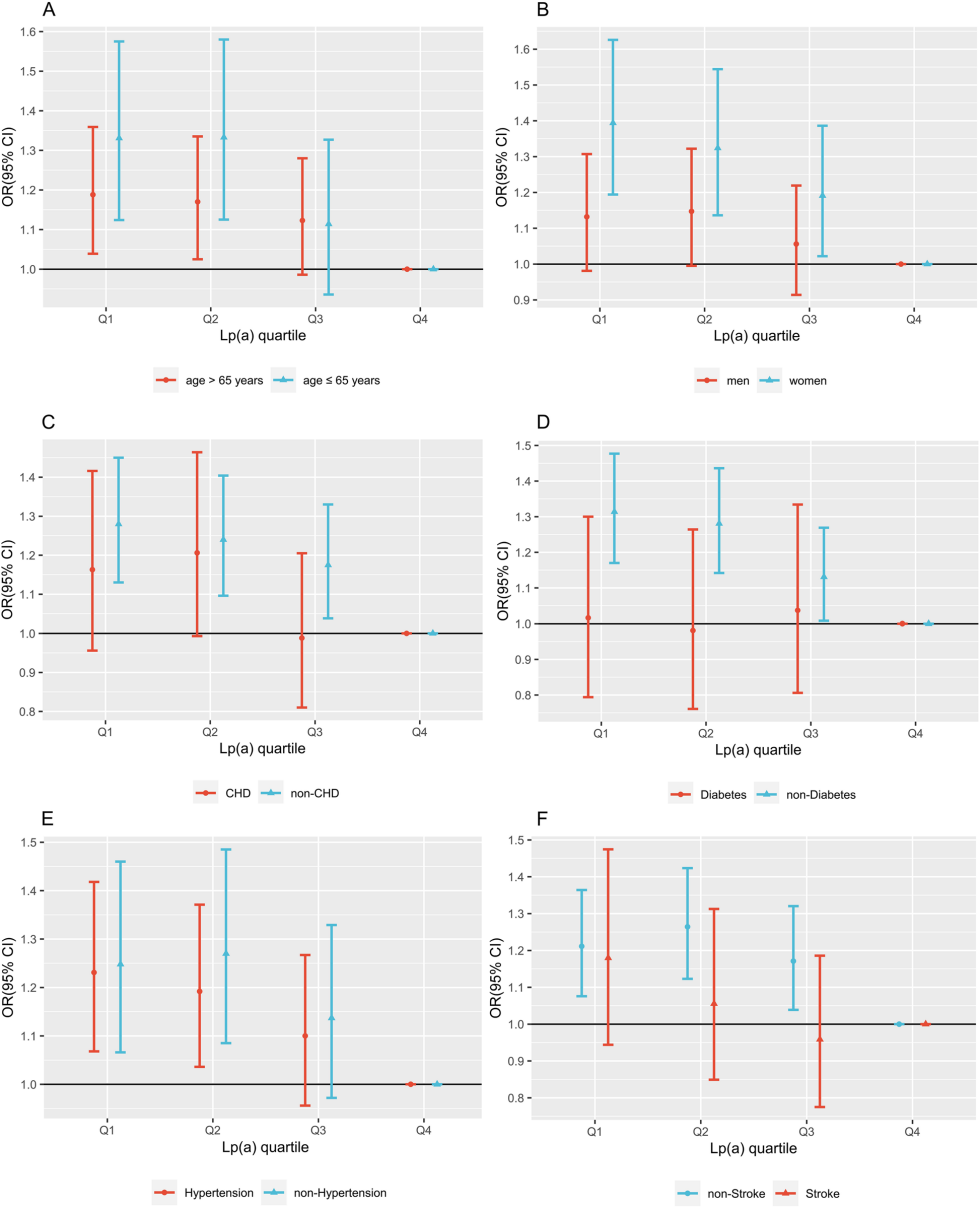
Odds ratios (95% CI) of Lp(a) quantiles stratified by age (**A**), sex (**B**), CHD (**C**), T2DM (**D**), hypertension (**E), **and ischemic stroke (**F**). All the models were adjusted for BMI, blood glucose, CRP, HCY, statin status, SBP, and TG

According to the stratified analysis of age (≤ 65 years and > 65 years), Q1 (under 65 years group: 1.331, [1.124–1.575], *P* < 0.001; over 65 years group: 1.188, [1.039–1.359], *P* < 0.001) and Q2 (under 65 years group: 1.333, [1.125–1.580]; *P* < 0.001, over 65 years group: 1.170, [1.025–1.335], *P* < 0.001) exhibited significant negative correlations with AF in both subgroups (Fig. 2A). According to the stratified analysis by sex, an inverse association was observed only to be statistically significant in women, Q1 (1.394 [1.194–1.626]; *P* < 0.001), Q2 (1.324 [1.136–1.544]; *P* < 0.001), and Q3 (1.191 [1.022–1.386]; *P* < 0.001) (Fig. 2B). However, significant relationships between Lp(a) and AF were not observed in patients with CHD, T2DM, or ischemic stroke (Fig. 2C, D, and F). Furthermore, the status of hypertension did not affect the significance of the Lp(a)-reduced elevated AF (Fig. 2E).

## Discussion

In our large-scale retrospective cohort study, a significant inverse correlation between AF and Lp(a) levels was found. Thus, an Lp(a) level lower than 32.42 mg/dL could be a potential risk factor for AF. Interestingly, an inverse relationship was found only in women. Among CHD patients and T2DM patients, the negative association was eliminated, indicating their confounding effect. In addition, hypertension status and age did not seem to influence the inverse relationship in the study population.

In comparison with published studies, a prospective community-based study with 10,127 participants without AF at baseline found no significant linear trend between AF and Lp(a) quantiles (HR, 0.98; 95% CI, [0.82–1.17]) [14]. A recent Mendelian randomization study based on the European population found a robust positive causal association [15]. These studies, however, did not recruit Asian participants. Compared to non-Hispanic Caucasians [median:12 (5–32)mg/dL], Hispanics [median:19 (8–43)mg/dL], and other ethnicities, Han ethnicity exhibits the lowest serum Lp(a) level [median: 11 (4-12)] mg/dL) [9]. In addition, patients whose Lp(a) is over 15 mg/dL can have a significantly elevated cardiac risk among Han ethnicity, which is notably lower than the recommended screening threshold by current global consensus (30 mg/dL or 50 mg/dL) [16]. Moreover, a published Mendelian study in the Han Chinese population showed that genetically elevated Lp(a) was inversely associated with the risk of atrial fibrillation [17]. In a multicenter study, a subgroup analysis of a Chinese population gave a similar result [18]. In sum, we consider that patients from different races may have different Lp(a) risk thresholds, which could influence the results.

The mechanism driving this relationship is unclear. The potential hypothesis may include the following: First, the inverse relationship between lipoproteins and AF may be due to its cholesterol cloud function in cell membrane stabilization, which can prevent abnormal discharge of cardiomyocytes [19, 20]. It was also found that cholesterol depletion disrupted the contractile function of cardiomyocytes [21]. Second, inflammation status may also be related to the relationship between the development of AF and lipid levels [22]. Studies have found that under inflammatory conditions, blood TC and LDL-C are reduced [22]. Our results verified this: AF group versus control: TC (4.09 *vs*. 4.63 mmol/L, *P* < 0.001) and LDL-C (2.35 *vs*. 2.70 mmol/L, *P* < 0.001). The AF group exhibited a higher median CRP level than the control in this study (4.03 *vs*. 3.67 mg/L, *P* < 0.001), which represented a higher inflammation status.

Beyond that, the negative correlation between elevated Lp(a) and AF was found only to be significant among women in this study (Fig. 2B). Sex differences in plasma lipid profiles have been well studied [23]. Similarly, the results from the BiomarCaRE Consortium showed that TC and other proatherogenic lipoproteins, such as Lp(a), are protective factors against AF, especially in women [24]. This study also showed similar results that women have significantly higher TC levels than men [4.65 (3.91, 5.46) *vs*. 4.29 (3.58, 5.00) mmol/L, *P* < 0.001]. Studies have found that hormones, insulin sensitivity, and body fat distribution may contribute to sex differences in the AF-lipoprotein relationship [25]. However, the Women’s Health Study in Switzerland reported that cholesterol-deficient small LDL particles are driving the negative association with AF, rather than cholesterol-rich LDL, such as Lp(a) [26]. Therefore, well-designed prospective clinical trials will confirm whether sex affects the correlation between Lp(a) and AF in the future.

The role of CHD in the AF-Lp(a) relationship is complicated. On the one hand, CHD and ischemic stroke are highly associated with Lp(a) [9]. On the other hand, the interaction between CHD and AF as a vicious cycle has been shown to have multiple mechanisms [27]. In this study, the inverse correlation disappeared among the patients with CHD. It is worth noting that CHD status was adjusted when including participants, and the proportions of CHD patients in the AF group and control group were 26.94% and 26.93%, respectively. In ischemic stroke patients, the AF-Lp(a) relationship was reversed in the Q3 group. A recent prospective cohort study from China found that high Lp(a) levels are associated with increased stroke recurrence [28]. A meta-analysis involving 41 studies also found that elevated Lp(a) increases ischemic stroke risk [29]. The positive association between stroke and Lp(a) may be the source of its influence on the result. However, prospective clinical trials are needed to confirm this in the future.

### Study strengths and limitations

Of note, this large-scale study is the first to focus on Lp(a) and AF relationships in the Chinese population. We designed well-constructed layered analysis models and performed subgroup analyses to support the conclusion and hypothesis. In addition, we searched for the most confounding factors between the Lp(a) concentration and AF, for instance, statin use [30] and CHD status, and then evaluated their effect to diminish the bias.

However, this study still has some limitations: (1) We are limited to assessing the correlation rather than any causal association as a retrospective cohort study. (2) This study is based on clinical data from a single hospital center; thus, possible bias could exist. (3) Recent studies found that the diameters of Lp(a) particles are determined by the numbers of kringle repeats in 6q26-27 chromosomal regions in the LPA gene, which vary greatly [31]. The particle diameter represents the particle size of lipoprotein; thus, the traditional mass index “mg/dL” cannot well evaluate the serum Lp(a) particle numbers. Considering the genetic heterogeneity among individuals simply converting the Lp(a) measurement units is not a wise choice. We hope to enhance the Lp(a) measuring methods in future studies**.**

## Conclusion

A significant inverse association was found, with lower circulating Lp(a) being related to elevated AF. However, this relationship appeared only in women and was not influenced by age or hypertension status; in CHD patients and T2DM patients, this inverse association was eliminated. This newly found association between blood Lp(a) and AF provides a novel perspective on the role of Lp(a) in AF patients, suggesting that Lp(a) may be useful for risk stratification of AF in female individuals. Therefore, among women, patients with Lp(a) concentrations below 32.42 mg/dL should stay alert to their potential AF.

## Supplementary Information

**Additional file 1: Table S1.** The correlation indices between Lp(a) and the variables. **Figure S1.** The distribution of Lp(a) concentration in all study population. **Figure S2.** Odd ratios (95% confidence intervals) for AF of Lp(a) quartiles stratified by hypertension status. This Model was adjusted for BMI, Blood glucose, CRP, HCY, statin status, SBP, and TG. **Figure S3.** Odd ratios (95% confidence intervals) for AF of Lp(a) quartiles stratified by statin use status. This Model was adjusted for BMI, Blood glucose, CRP, and SBP. **Figure S4.** Odd ratios (95% confidence intervals) for AF of Lp(a) quartiles stratified by stroke status. This Model was adjusted for BMI, Blood glucose, CRP, HCY, SBP and statin status. **Figure S5.** Odd ratios (95% confidence intervals) for AF of Lp(a) quartiles stratified by total cholesterol. This Model was adjusted for BMI, Blood glucose, CRP, HCY, SBP and statin status. **Figure S6.** Odd ratios (95% confidence intervals) for AF of Lp(a) quartiles stratified by total triglycerides. This Model was adjusted for BMI, Blood glucose, CRP, HCY, SBP and statin status. **Figure S7.** The absolute standardized mean (ASD) differences before and after propensity score matching.

## Data Availability

Participant materials cannot be made public because they contain information that could compromise the privacy of the study participants, but the corresponding authors may provide a minimum amount of data upon reasonable request.
