# Correction: Low lipoprotein(a) concentration is associated with atrial fibrillation: A large retrospective cohort study

**DOI:** 10.1186/s12944-023-01805-3

**Published:** 2023-03-11

**Authors:** Junjie Tao, Xinlei Yang, Qingkai Qiu, Feng Gao, Wenchong Chen, Lijuan Hu, Yuan Xu, Yingping Yi, Hui Hu, Long Jiang

**Affiliations:** 1 Department of Cardiovascular Medicine, The Second Affiliated Hospital of Nanchang University, Nanchang, Jiangxi Province China; 2 Department of Clinical Medical, The Second Clinical Medical College of Nanchang University, Nanchang, Jiangxi Province China; 3 Department of Biobank Center, The Second Affiliated Hospital of Nanchang University, Nanchang, Jiangxi Province China; 4 Department of Nursing, Nanchang Medical College, Nanchang, Jiangxi Province China; 5 Department of Medical Big Data Center, The Second Affiliated Hospital of Nanchang University, Nanchang, Jiangxi Province China

**Correction: Lipids Health Dis 21, 119 (2022)**

**https://doi.org/10.1186/s12944-022-01728-5**

Following publication of the original article [1], the authors requested to correct Long Jiang’s affiliation to:

1. Department of Cardiovascular Medicine, The Second Affiliated Hospital of Nanchang University, Nanchang, Jiangxi Province, China.
2. Department of Clinical Medical, The Second Clinical Medical College of Nanchang University, Nanchang, Jiangxi Province, China.

The original article has been updated.
