# Supplementary material for: Low lipoprotein(a) concentration is associated with atrial fibrillation: a large retrospective cohort study
Source: Lipids Health Dis. 2022 Nov 14;21:119. doi: 10.1186/s12944-022-01728-5 (PMC9661736; doi:10.1186/s12944-022-01728-5)
Supplement: Supplementary file 1 — Additional file 1: Table S1. The correlation indices between Lp(a) and the variables. Figure S1. The distribution of Lp(a) concentration in all study population. Figure S2. Odd ratios (95% confidence intervals) for AF of Lp(a) quartiles stratified by hypertension status. This Model was adjusted for BMI, Blood glucose, CRP, HCY, statin status, SBP, and TG. Figure S3. Odd ratios (95% confidence intervals) for AF of Lp(a) quartiles stratified by statin use status. This Model was adjusted for BMI, Blood glucose, CRP, and SBP. Figure S4. Odd ratios (95% confidence intervals) for AF of Lp(a) quartiles stratified by stroke status. This Model was adjusted for BMI, Blood glucose, CRP, HCY, SBP and statin status. Figure S5. Odd ratios (95% confidence intervals) for AF of Lp(a) quartiles stratified by total cholesterol. This Model was adjusted for BMI, Blood glucose, CRP, HCY, SBP and statin status. Figure S6. Odd ratios (95% confidence intervals) for AF of Lp(a) quartiles stratified by total triglycerides. This Model was adjusted for BMI, Blood glucose, CRP, HCY, SBP and statin status. Figure S7. The absolute standardized mean (ASD) differences before and after propensity score matching. [file 12944_2022_1728_MOESM1_ESM.docx]

**Low lipoprotein(a) concentration is associated with atrial fibrillation: A large retrospective cohort study**

**Short title:** Lp(a) levels and atrial fibrillation

Junjie Tao^1,2#^, Xinlei Yang^3#^, Qingkai Qiu ^1,2^, Feng Gao ^1,2^, Wenchong Chen ^1,2^, Lijuan Hu ^4^, Yuan Xu ^5^, Yingping Yi ^5^, Hui Hu ^6*^, Long Jiang ^2*^

^1^ Department of Cardiovascular Medicine, the Second Affiliated Hospital of Nanchang University, Nanchang, Jiangxi, China

^2^ The Second Clinical Medical College of Nanchang University, The Second Affiliated Hospital of Nanchang University，Jiangxi, Nanchang 330006, China

^3^ Biobank center, the Second Affiliated Hospital of Nanchang University

^4^ Department of Nursing, Nanchang Medical College, Nanchang, Jiangxi, China

^5^ Department of Medical Big Data Center, the Second Affiliated Hospital of Nanchang University, Nanchang, Jiangxi, China

^6^ Medical big data center, the Second Affiliated Hospital of Nanchang University, Nanchang, Jiangxi, China

***Correspondence to:** Long Jiang, Department of Cardiovascular Medicine, the Second Affiliated Hospital of Nanchang University, Nanchang, Jiangxi, China; e-mail: [skyiadx@hotmail.com](mailto:skyiadx@hotmail.com); Tel: +86-13767026990. Hui Hu, Medical big data center, the Second Affiliated Hospital of Nanchang University, Nanchang, Jiangxi, China; email: [huhuillz@sina.com](mailto:huhuillz@sina.com); Tel: +86-13576978125.

Other authors information: Junjie Tao, email: [4203119304@email.ncu.edu.cn](mailto:4203119304@email.ncu.edu.cn). Xinlei Yang, Biobank center, the Second Affiliated Hospital of Nanchang University; email: [yxl2251259@163.com](mailto:yxl2251259@163.com); Qingkai Qiu: [2339816616@qq.com](mailto:2339816616@qq.com). Feng Gao: The Second Clinical Medical College of Nanchang University, the Second Affiliated Hospital of Nanchang University, Jiangxi, Nanchang; email: 1400954354@qq.com. Wenchong Chen, email: 3139691557@qq.com. Lijuan Hu, email: hlj2008happy@163.com. Yuan Xu, email: xuyuan0805@126.com. Yingping Yi, email: yyp66@126.com.

**Supplements**

**Table S1.** The correlation indices between Lp(a) and the variables.

| **Characteristics** | **r** | **P-value** |
| --- | --- | --- |
| **Demographic data** |  |  |
| Sex | -0.020 | 0.015 |
| Ages | 0.042 | <0.001 |
| BMI | -0.0408 | <0.001 |
| Smoker | -0.0209 | 0.014 |
| Alcohol taking status | -0.0191 | 0.928 |
| SBP | <0.0001 | 0.985 |
| DBP | -0.0109 | 0.204 |
| **Medical history** |  |  |
| Atrial fibrillation | -0.030 | <0.001 |
| Ischemic stroke | 0.023 | 0.007 |
| Type 2 diabetes mellitus | -0.013 | 0.113 |
| Coronary heart disease | 0.037 | <0.001 |
| Hypertension | 0.009 | 0.286 |
| **Medication on admission** |  |  |
| β-receptor antagonists | 0.008 | 0.305 |
| Statin | 0.066 | <0.001 |
| **Laboratory values** |  |  |
| LDL-C | 0.136 | <0.001 |
| Apo(a) | 0.022 | 0.007 |
| Apo(b) | 0.133 | <0.001 |
| HDL-C | 0.113 | <0.001 |
| TC | 0.120 | <0.001 |
| TG | -0.037 | <0.001 |
| HCY | -0.010 | 0.241 |
| Albumin | -0.033 | <0.001 |
| CRP | 0.030 | <0.001 |
| Blood glucose | -0.008 | 0.349 |
| Uric acid | -0.015 | 0.071 |
| Serum creatine | 0.034 | <0.001 |

Continuous variables exhibited as median [25, 75%]. Apo(a), apolipoprotein A; Apo(b), apolipoprotein B; BMI, body mass index; CRP, C-reactive protein; DBP, diastolic blood pressure; HDL-C, high-density lipoprotein cholesterol; HCY, homocysteine; LDL-C, low-density lipoprotein cholesterol; SBP, systolic blood pressure; TC, total cholesterol; TG, triglyceride.

**
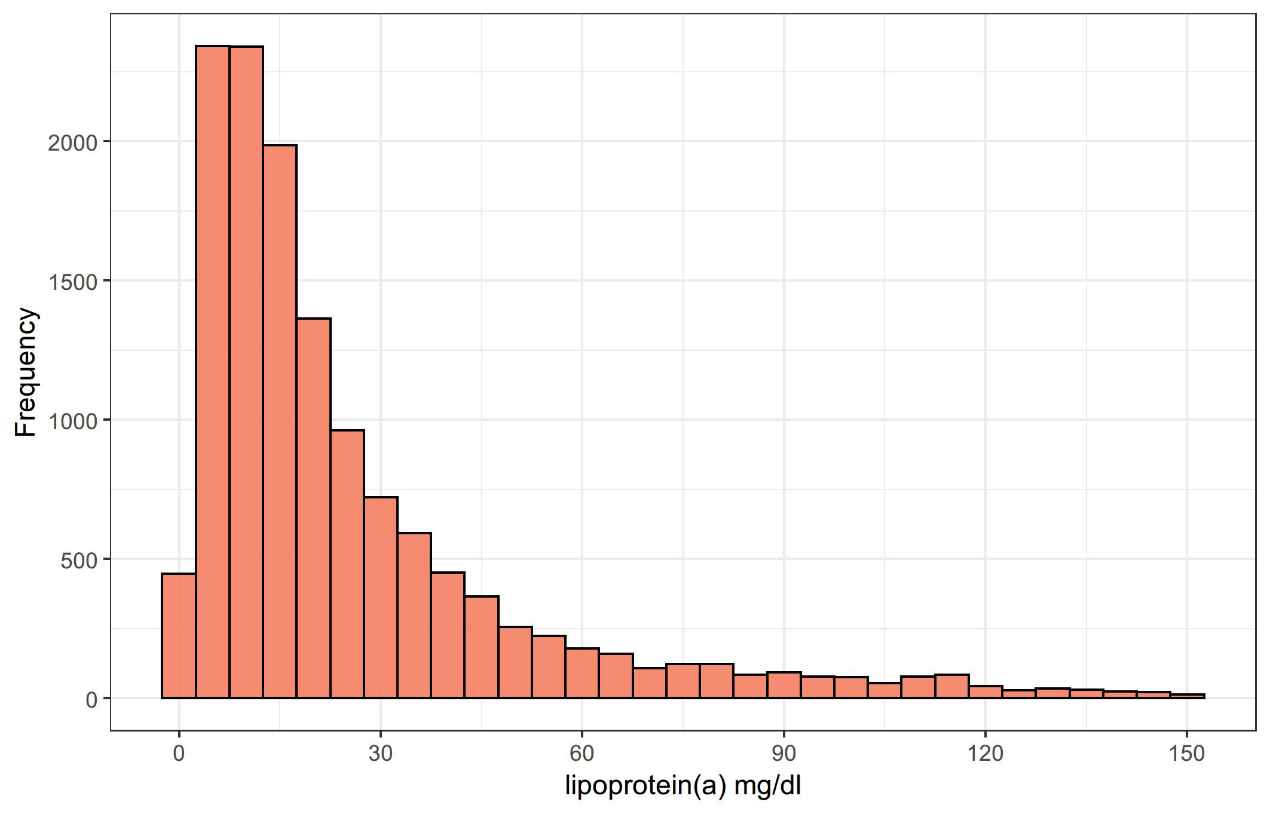
**

**Figure S1.** The distribution of Lp(a) concentration in all study population.


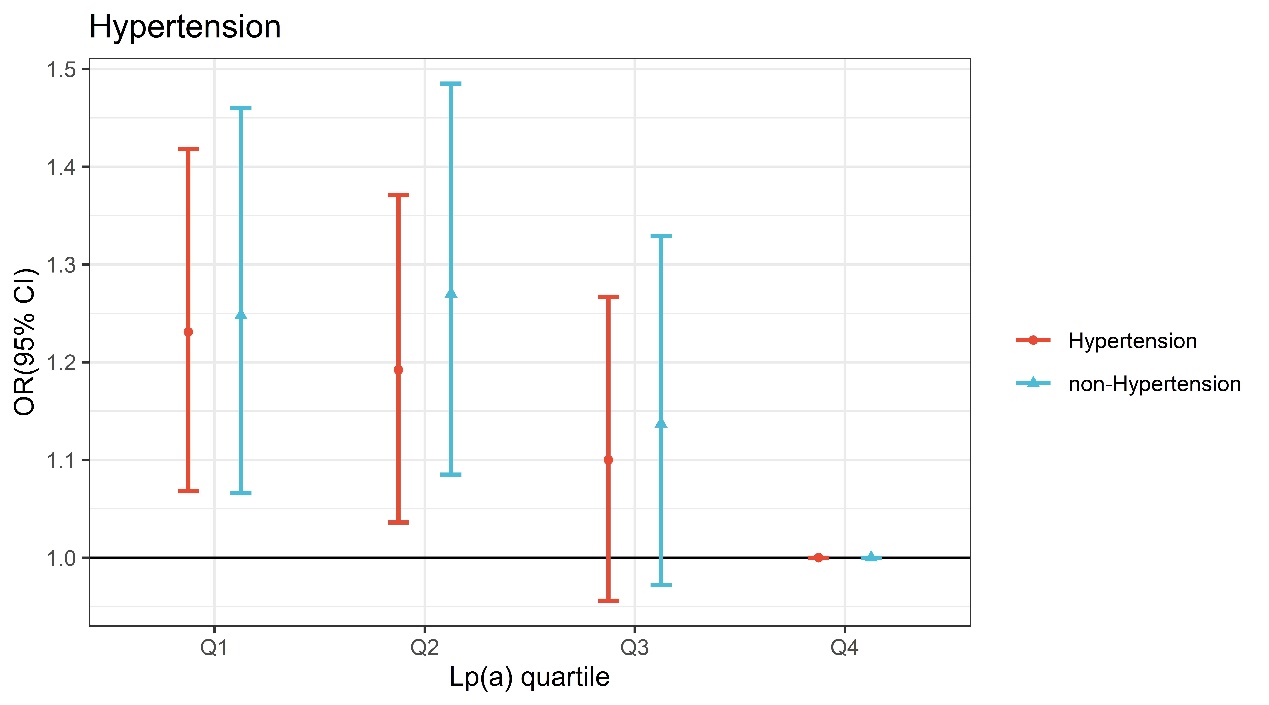


**Figure S2.** Odd ratios (95% confidence intervals) for AF of Lp(a) quartiles stratified by hypertension status. This Model was adjusted for BMI, Blood glucose, CRP, HCY, statin status, SBP, and TG.


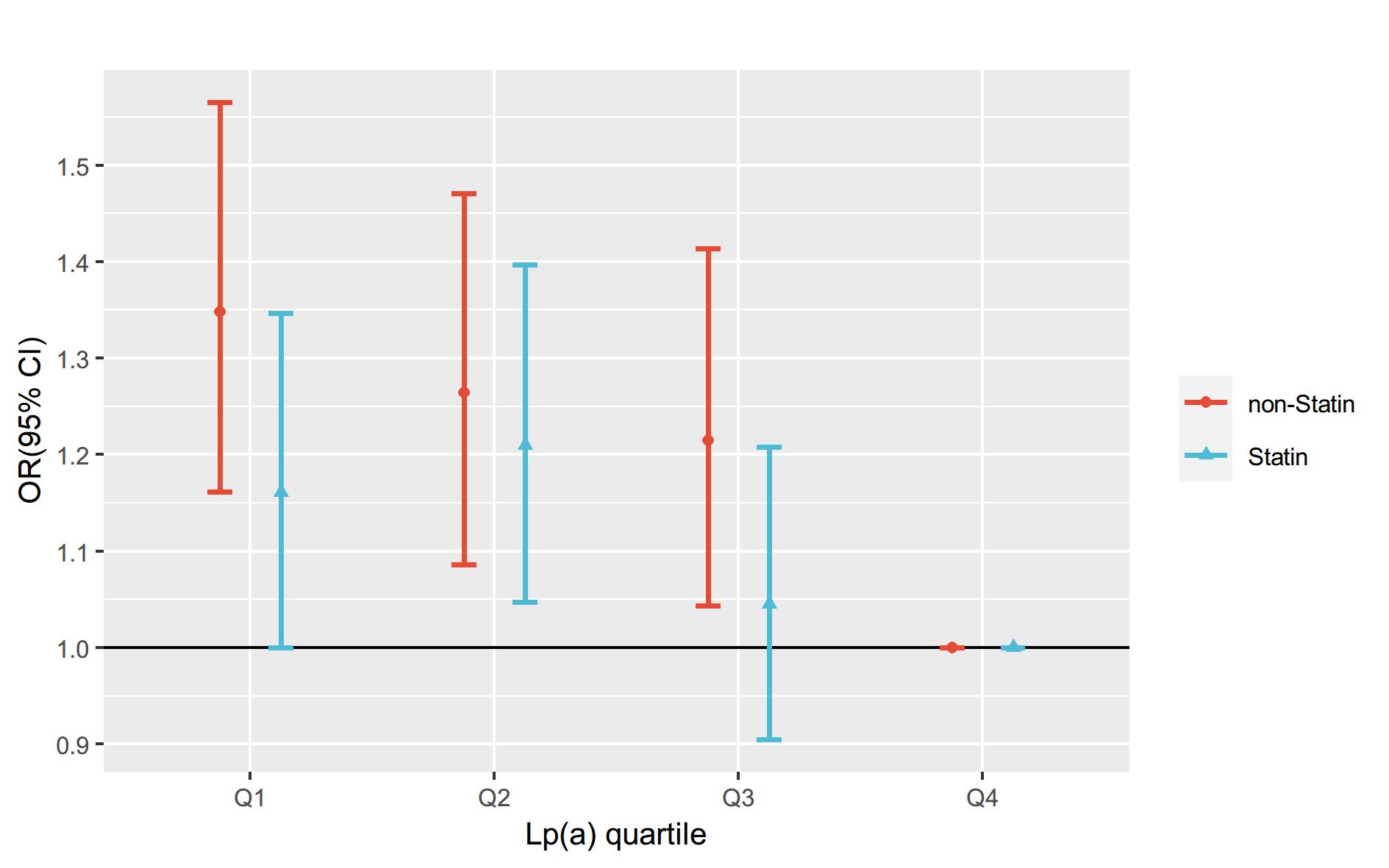


**Figure S3.** Odd ratios (95% confidence intervals) for AF of Lp(a) quartiles stratified by statin use status. This Model was adjusted for BMI, Blood glucose, CRP, and SBP.


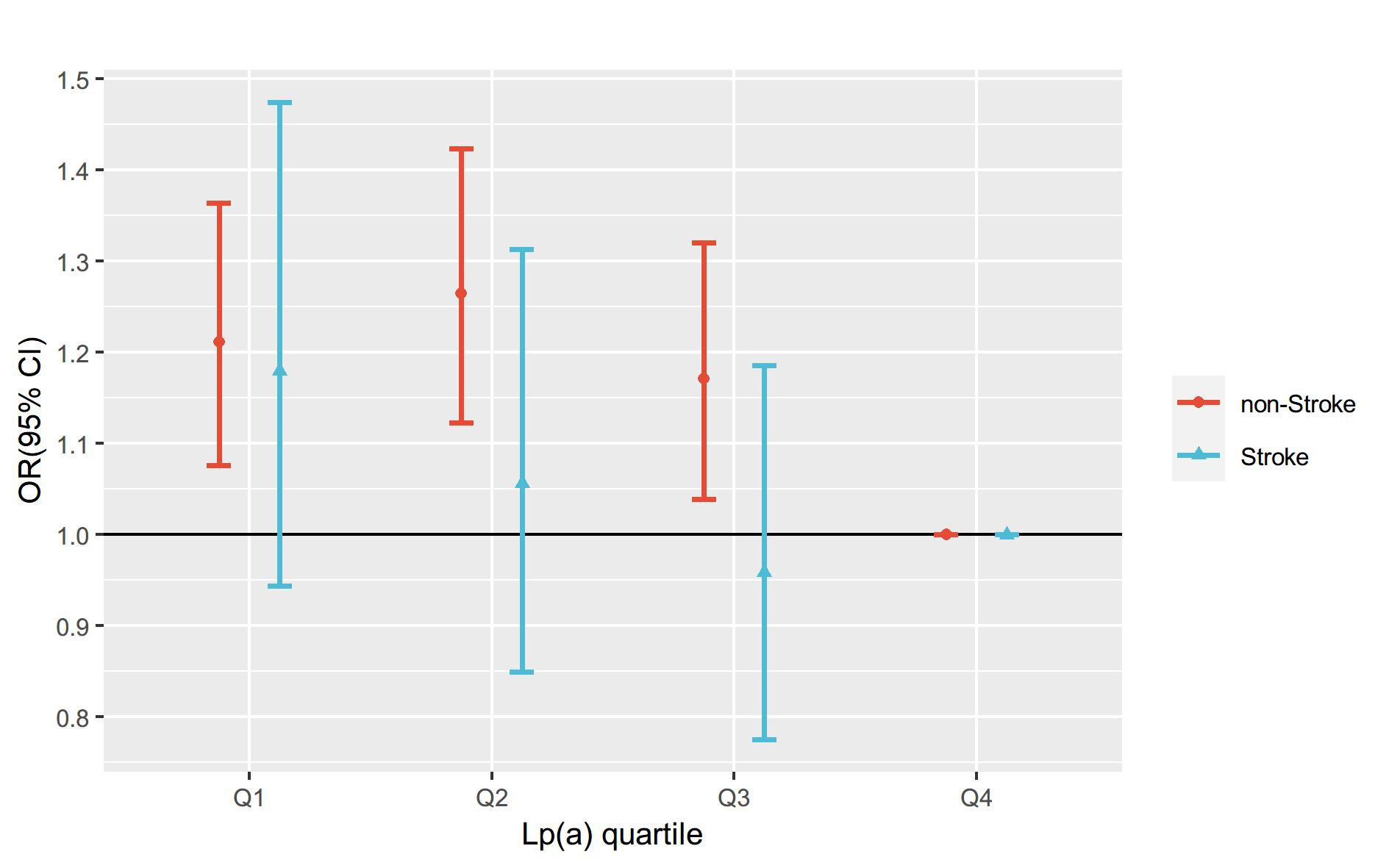


**Figure S4.** Odd ratios (95% confidence intervals) for AF of Lp(a) quartiles stratified by stroke status. This Model was adjusted for BMI, Blood glucose, CRP, HCY, SBP and statin status.


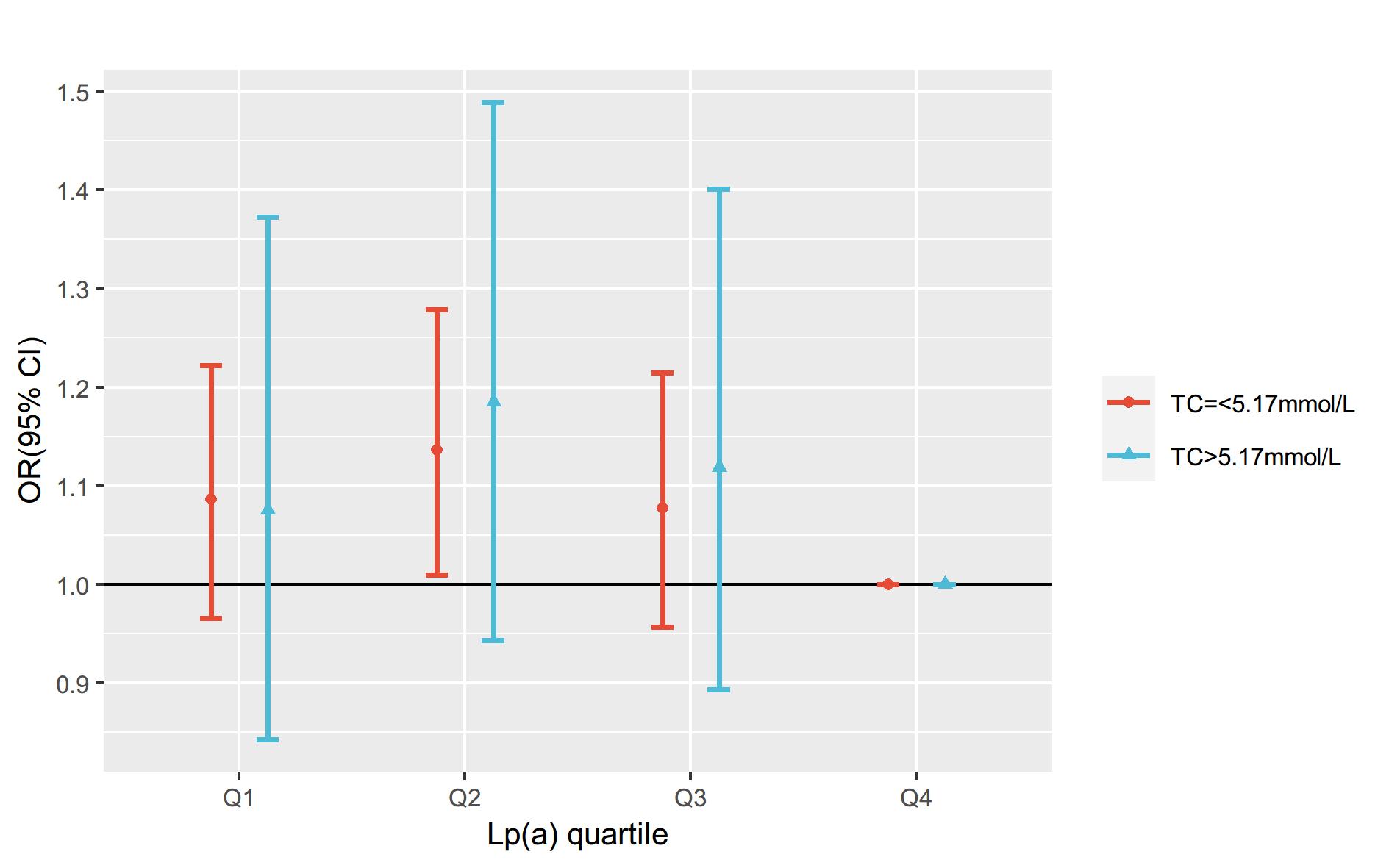


**Figure S5.** Odd ratios (95% confidence intervals) for AF of Lp(a) quartiles stratified by total cholesterol. This Model was adjusted for BMI, Blood glucose, CRP, HCY, SBP and statin status.


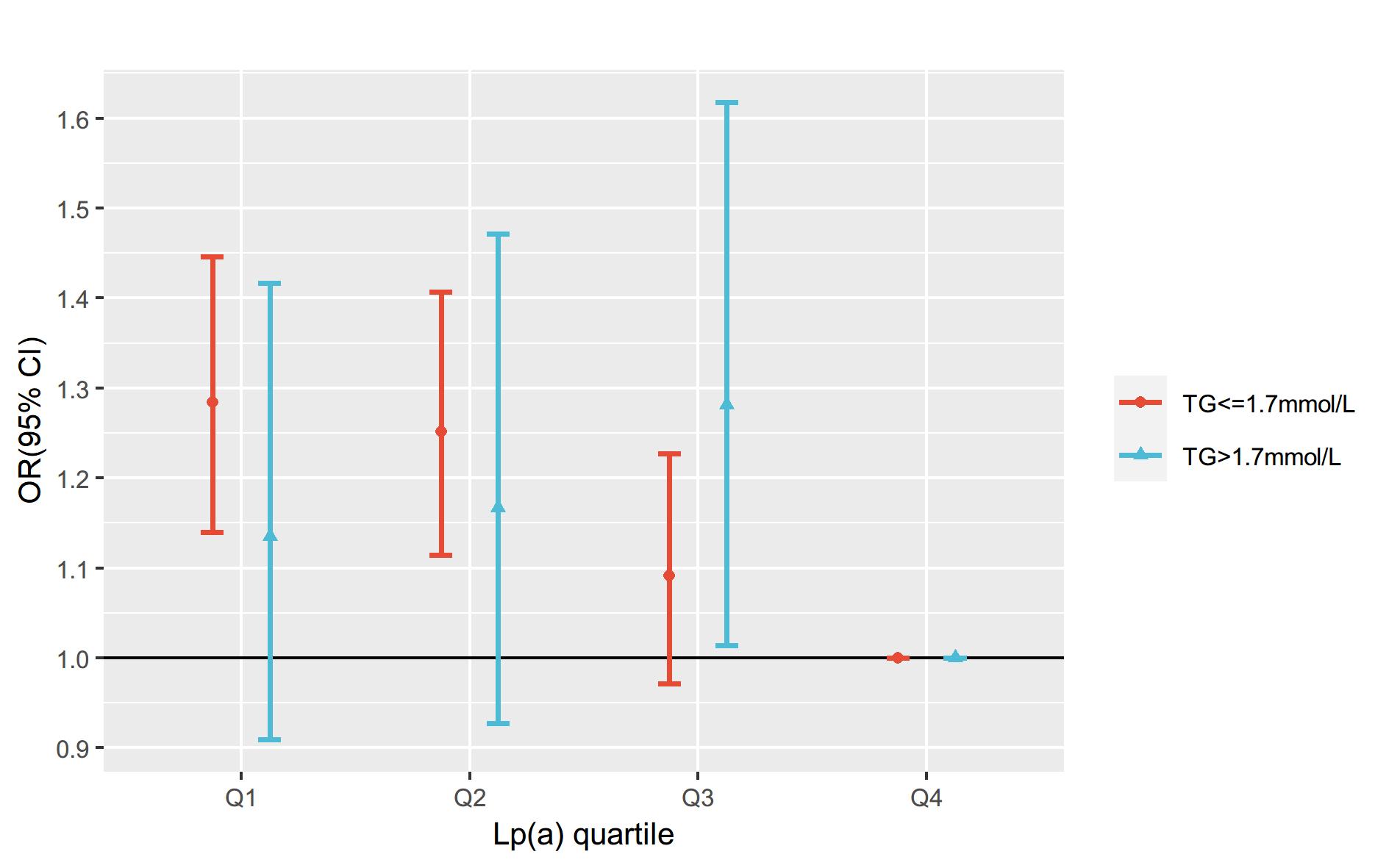


**Figure S6.** Odd ratios (95% confidence intervals) for AF of Lp(a) quartiles stratified by total triglycerides. This Model was adjusted for BMI, Blood glucose, CRP, HCY, SBP and statin status.


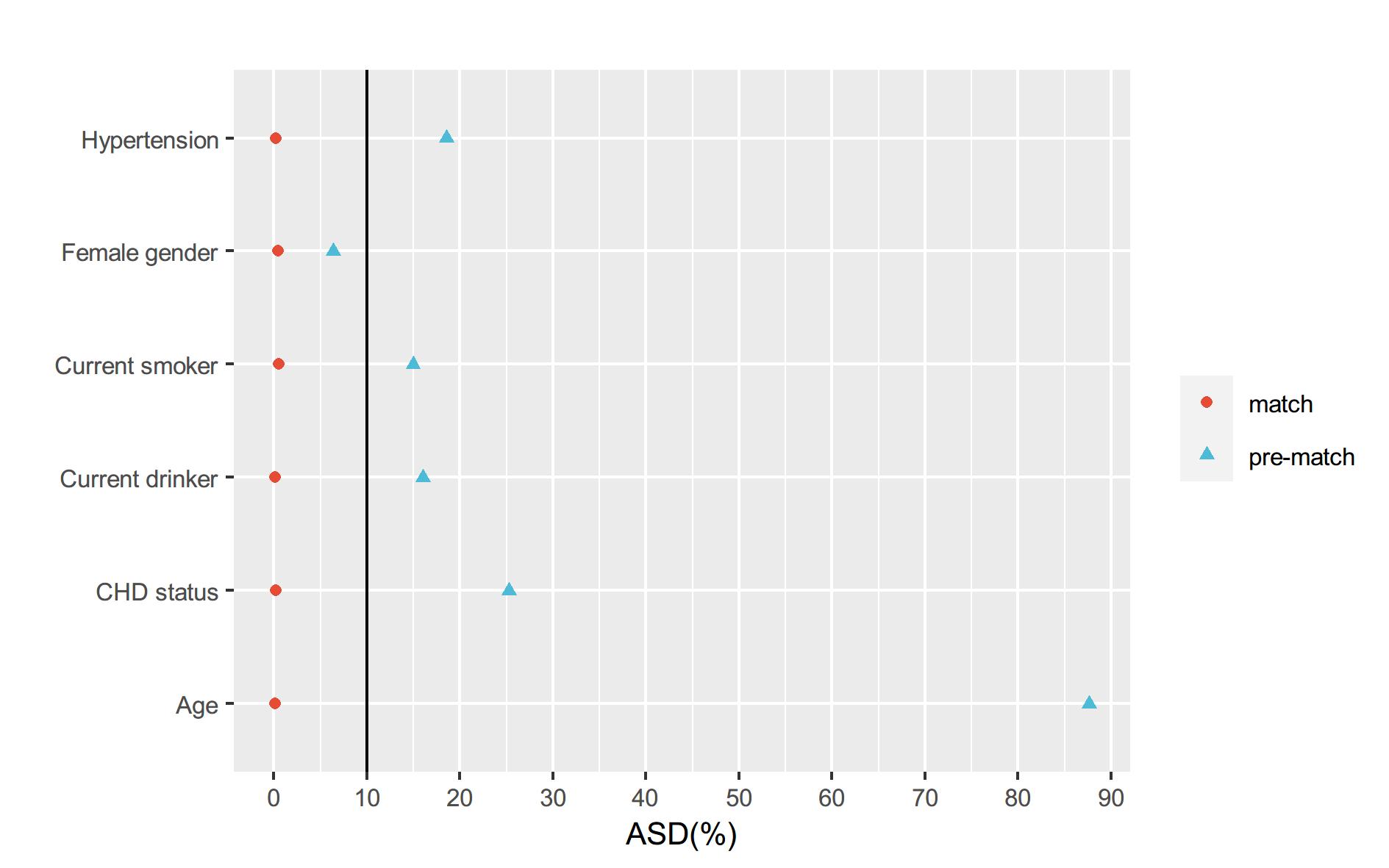


**Figure S7.** The absolute standardized mean (ASD) differences before and after propensity score matching.
